# Genome sequence of cluster F1 *Mycobacterium smegmatis* phage Fastidio

**DOI:** 10.1128/mra.00148-25

**Published:** 2025-03-19

**Authors:** Matthew N. Jackson, Hyunbi Hwang, Payson C. Danielson, Ethan Walbom, Jayden Longhurst, Bartel Van Oostendorp, Christopher C. Harrell, Austin M. Johnson, Natalie A. Olsen, Parker Danielson, Thomas Wilhite, Jeffrey K. Schachterle, Staci Avery, Donald P. Breakwell, Brett E. Pickett

**Affiliations:** 1 Department of Microbiology and Molecular Biology, Brigham Young University 723033, Provo, Utah, USA; Department of Biology, Queens College, Queens, New York, USA

**Keywords:** bacteriophages, genome analysis

## Abstract

Fastidio is an F1 subcluster bacteriophage in the *Caudoviricetes* class, infecting *Mycobacterium smegmatis* strain mc²155. The genome is 55,839 base pairs in length and contains several putative novel open reading frames. The isolation, annotation, and analysis of Fastidio, together with other bacteriophages, improve our knowledge of phage diversity.

## ANNOUNCEMENT

Fastidio was isolated from a soil sample collected in Provo, UT, USA (GPS: 40.214582 N, 111.618295 W). The sample was suspended in 7H9 broth and filtered through a 0.22 µm filter. An aliquot was used to infect host cells, mixed with top agar and then plated on 7H10 agar prior to incubation at 37°C. Resulting plaques were picked using a sterile micropipette tip, and high-titer lysates were prepared following three rounds of purification. High-titer lysate was sent to CD Genomics (Beverly, NY, USA), where 0.5 µg DNA was extracted using the Phage DNA Isolation Kit (Norgen) protocol prior to DNA sequencing. NEBNext Ultra DNA Library Prep Kit for Illumina (NEB, USA) was used following the manufacturer’s recommendations, and indices were used. DNA samples were sonicated to 350 bp, end-polished, A-tailed, and ligated with the full-length adaptor for sequencing. The product was purified (AMPure XP system) and quantified with an Agilent 2100 Bioanalyzer and qPCR. Approximately 1.3 million 150-bp paired-end reads were generated using the Illumina NovaSeq X instrument. TrimGalore was applied with a minimum length of 20 bases and a minimum Phred score of 20 (1). Then, *de novo* assembly of the reads was performed using CONSED 2.9 (2) with Unicycler version 0.5.0 (3), resulting in a 55,839 base pair genome (3,593× coverage) with 61.6% G + C content.

**Fig 1:**
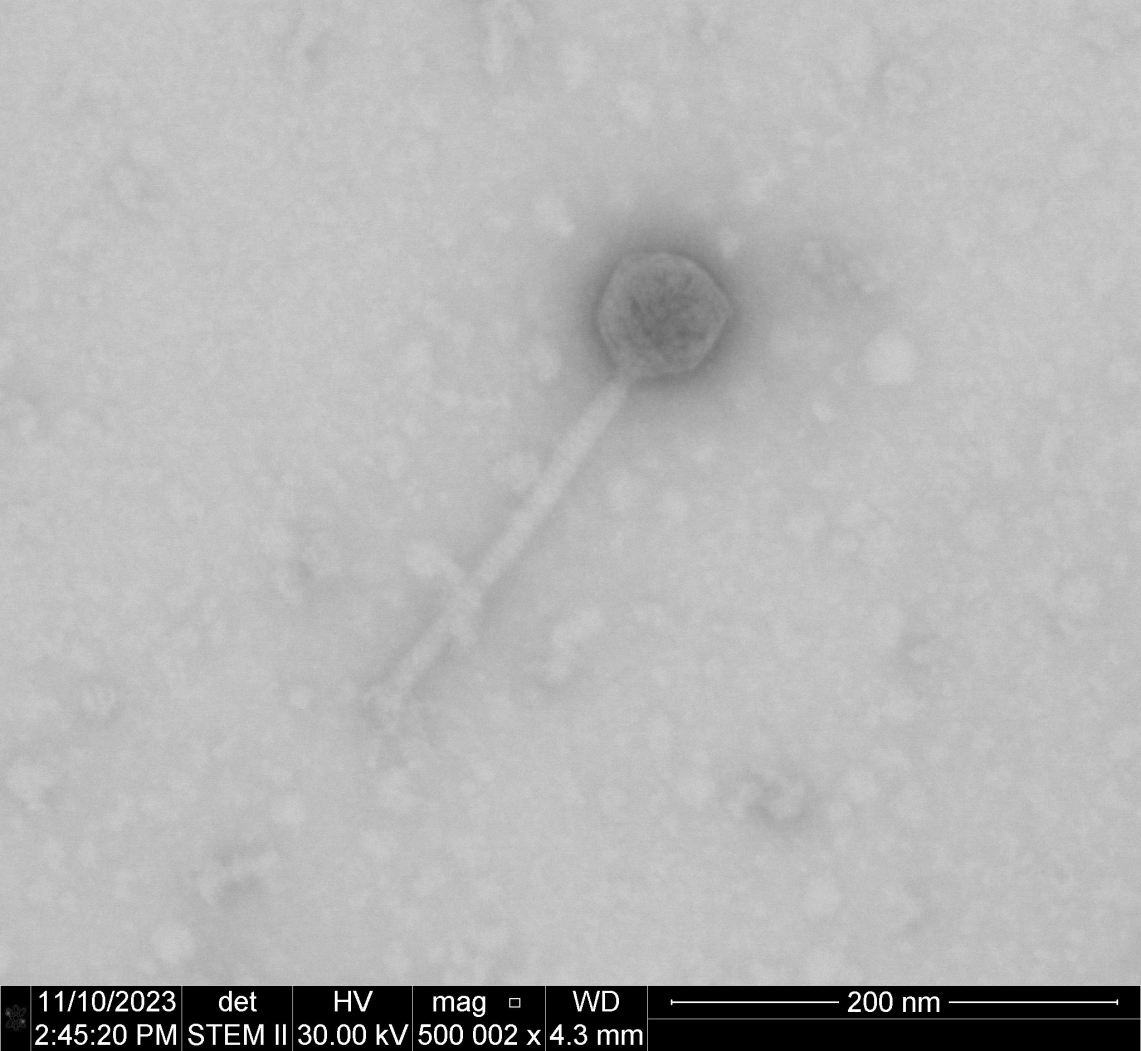
Negative-stained transmission electron micrograph of the Fastidio phage (500,000× magnification and 20 kV accelerating voltage, Tecnai TF-20).

Fastidio is predicted to be a temperate phage by cluster sequence homology and has siphovirus-like morphology. It has a long, non-contractile tail with an icosahedral head (Fig. 1), which is characteristic of the *Caudoviricetes* class. This phage generally forms cloudy plaques within 2–3 days of isolation. The genome is predicted to replicate through a 10 base pair 3′ sticky overhang. The Fastidio phage was assigned to the F1 phage subcluster by phagesDB (4) (https://phagesdb.org/) through best-match nucleotide sequence homology searches.

The genome of *Fastidio* was initially auto-annotated using Glimmer version 3.0 and GeneMark 2.5 (5, 6) and then refined in DNA Master (7). Briefly, this refinement entailed using Starterator, Phamerator (8), PhageScope (9), tRNAscanSE (10), HHPRED (11), and BLASTp (12) (with default parameters) to capture data that support the presence, location, and start/stop sites for putative open reading frames using criteria specified by the Science Education Alliance-Phage Hunters Advancing Genomics and Evolutionary Science program. The genome contained 105 putative protein-coding genes and a single tRNA gene. We also compared genes across the F1 subcluster and identified a total of three potentially novel open reading frames in this genome, including gp26, gp100, and gp105.

## Data Availability

The sequence for this Fastidio phage is publicly available in GenBank with accession number PQ244011. The raw sequencing reads are available in the Sequence Read Archive (SRA) with accession number SRX27013404.
